# Association between PD‐1 inhibitor‐related adverse events and frailty assessed by frailty index in lung cancer patients

**DOI:** 10.1002/cam4.5669

**Published:** 2023-02-02

**Authors:** Jun Li, Xiaolin Zhang, Shuang Zhou, Ying Zhou, Xinmin Liu

**Affiliations:** ^1^ Department of Geriatrics Peking University First Hospital Beijing People's Republic of China; ^2^ Department of Pharmacy Peking University First Hospital Beijing People's Republic of China

**Keywords:** frailty, frailty index, immune‐related adverse events, lung cancer, PD‐1 inhibitor

## Abstract

**Background:**

The programmed cell death protein 1 (PD‐1) inhibitor, as one of the immune checkpoint inhibitors (ICIs), is the standard treatment for advanced lung cancer. However, immune‐related adverse events (irAEs) remain poorly understood toxicities. It is unclear whether frailty plays a role in the occurrence of irAEs. Thus, we assess whether irAEs occur more often in frail patients than in non‐frail patients according to the Frailty Index (FI).

**Methods:**

A retrospective study was conducted. Medical records from lung cancer patients treated with PD‐1 inhibitors (Sintilimab, Camrelizumab, Tislelizumab, and Pembrolizumab) at Peking University First Hospital (May 2018–June 2022). Patients were categorized into non‐frail and frail groups according to a cut‐point of 0.25 by FI. The FI calculation included 28 baseline variables, all of which were health deficits measured by questionnaires and body measurements.

**Results:**

The statistical analysis included 114 advanced lung cancer patients. The median age was 66 years, and the male/female ratio was 4.7:1 (94/20). Approximately 39 (34%) were classified as frail. PD‐1 inhibitor‐related adverse events occurred in 17.5% of patients, and 6.1% experienced irAEs of grade ≥3. There was no significant difference in the occurrence of irAEs (14.7% vs. 23.1%, *p* = 0.26), grade ≥ 3 irAEs (5.3% vs. 7.7%, *p* = 0.93), and treatment discontinuation due to irAEs (12.0% vs. 17.9%, *p* = 0.39) between non‐frail and frail patients. However, frail patients are more likely to have more than one type of irAEs and are more possibly to have checkpoint inhibitor pneumonitis (CIP) than non‐frail patients when they use PD‐1 inhibitors (*p* < 0.05). Frail patients had a longer hospital stay (6 vs. 3 days, *p* = 0.01).

**Conclusions:**

Frailty is not associated with severe irAEs, but is related to CIP. Meanwhile, it predicts more than one type of irAEs and a longer hospital stay. Frailty screening has added value to the decision‐making process for frail patients eligible for PD‐1 inhibitors.

## Graphical abstract

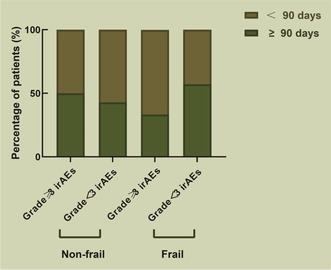

## 1 INTRODUCTION

The programmed cell death protein 1 (PD‐1) inhibitor, as an immune checkpoint inhibitors (ICIs), has become the first‐line therapy in advanced stages of different tumor types^1^, ^2^ and is increasingly used in early‐stage disease settings, including advanced lung cancer.^3^ ICIs, especially PD‐1 inhibitors, which improve outcomes for advanced lung cancer,^4^ are more tolerable than conventional chemotherapy.^5^, ^6^ However, PD‐1 inhibitor toxicity cannot be ignored. PD‐1 inhibitors can lead to an excessive inflammatory response, termed immune‐related adverse events (irAEs), involving any organs.^7^, ^8^ IrAEs can be mild, potentially severe, or even fatal. Therefore, these toxicities must be detected early and managed appropriately.

ICIs are considered a tolerable treatment option at an older age due to their favorable safety profile.^9^ Current data suggest that the incidence of irAEs does not increase with age.^10^ However, in these trials, the analysis is based on the patient's chronological age, and the relationship between the occurrence of irAEs and patients of different ages whose biological age exceeds their chronological age is unclear. Aging accelerates when the biological age exceeds chronological age. It is also associated with high morbidity, mortality, and reduced life expectancy.^11^ The potential impact of aging on immunotherapy efficacy and tolerability is unknown. The frailty index (FI) is a widely used measure of biological age.^12^ Fan et al. constructed a 28‐item FI as a proxy for chronological age to identify accelerated aging populations that might help to prevent premature death and extend life expectancy.^13^ The 28‐item FI applies to patients of all ages, not just the elderly. Consequently, based on the FI, we intend to establish a 28‐item FI including basic diseases, dysfunction, autonomous living ability, physiological indicators, and others (number of long‐term medications ≥5) to estimate whether non‐frail and frail patients are associated with a high risk of irAEs.

The real‐world clinical practice data on frailty patients treated with ICIs are woefully inadequate. To our knowledge, the predictive value of FI for irAEs has never been evaluated in lung cancer patients. Therefore, based on a real‐world cohort, this study aims to assess whether irAEs and its sequelae are more common in frail patients than in non‐frail patients using FI.

## 2 MATERIALS AND METHODS

### 2.1 Study Population and Data Collection

The electronic medical records of 114 patients were retrospectively reviewed with metastatic or unresectable advanced lung cancer who received PD‐1 inhibitors (Pembrolizumab, Sintilimab, Camrelizumab, and Tislelizumab) from May 2018 to June 2022 at Peking University First Hospital. Data from these patients were followed continuously until June 1, 2019. None of the patients had a history of previous ICI treatment and interstitial lung disease prior to treatment with the PD‐1 inhibitors. Patient's demographic and clinical characteristics, including age, sex, body mass index (BMI), Eastern Cooperative Oncology Group (ECOG) performance status, tumor type, type of PD‐1 inhibitors, combination medication, lymphocyte counts, albumin, neutrophil/lymphocyte ratio, grade ≥ 3 irAEs, clinically relevant irAEs, time to onset of irAEs, use of corticosteroids, treatment discontinuation due to irAEs, hospitalization due to irAEs, hospitalization duration, Intensive Care Unit (ICU) admission, remission of irAEs, and PD‐1 treatment restart, were collected. The proportion of patients who continued treatment with PD‐1 inhibitors for ≥90 days was analyzed to verify tolerance in both groups. Patients were followed up for 30, 60, 90, and 120 days after the diagnosis of irAE. This study was approved by the Research Ethics Committee of Peking University First Hospital.

The FI was calculated using 28 baseline variables, including basic illness (hypertension, coronary atherosclerotic cardiopathy, myocardial infarction, diabetes mellitus, stroke/transient ischemic attack, chronic obstructive pulmonary disease, bronchial asthma, tuberculosis, peptic ulcer, chronic cardiac insufficiency, benign prostatic hyperplasia, rheumatoid arthritis, chronic kidney disease, osteoporosis/fractures, and tumor), dysfunction (anxiety, depression, somnipathy, and cognitive dysfunction), ability to live independently (eating, urination, diaphoresis, bathing, and acting ability), physiological indicators (weight loss in one year, body‐mass index, albumin, neutrophil‐lymphocyte ratio (NLR)), and other aspects (multiple medications). These were health status deficits measured using questionnaires and physical examinations. FI is the proportion of an individual whose value is unhealthy among all health measures. FI = the number of unhealthy indicators in the health indicators/the number of health indicators. Two levels of frailty status, non‐frail (frailty index <0.25), and frail (FI ≥ 0.25), were defined (Table 1).

**TABLE 1:** Frailty scoring system.

	Health deficiencies	Score (Yes)	No
**Basic illness**			
1	Hypertension	1.0	0
2	Coronary atherosclerotic cardiopathy	1.0	0
3	Myocardial infarction	1.0	0
4	Diabetes mellitus	1.0	0
5	Stroke/ Transient ischemic attack	1.0	0
6	Chronic obstructive pulmonary disease	1.0	0
7	Bronchial asthma	1.0	0
8	Tuberculosis	1.0	0
9	Peptic ulcer	1.0	0
10	Chronic cardiac insufficiency	1.0	0
11	Benign prostatic hyperplasia	1.0	0
12	Rheumatoid arthritis	1.0	0
13	Chronic kidney disease	1.0	0
14	Osteoporosis/Fractures	1.0	0
15	Tumor	1.0	0
**Dysfunction**			
16	Anxiety and depression	1.0	0
17	Somnipathy	1.0	0
18	Cognitive dysfunction	1.0	0
**Ability to live independently**			
19	Take food	Completely independent: 0; Need help: 0.5; Absolute dependence: 1.0	
20	Urination	Completely independent: 0; Need help: 0.5; Absolute dependence: 1.0	
21	Diaphoresis	Completely independent: 0; Need help: 0.5; Absolute dependence:1.0	
22	Take a bath	Completely independent: 0; Need help: 0.5; Absolute dependence: 1.0	
23	Activity ability	Daily activities are not limited: 0; Daily activities partially limited: 0.25; Bedside activities: 0.5; Full bed rest: 1.0	
**Physiological indicators**			
24	Weight loss in one year (kg)	≥2.5 = 1.0; <2.5 = 0	
25	Body‐mass index (kg/m^2^)	<18.5 or ≥28 = 1.0; ≥24 and <28 = 0.5; ≥18.5 and <24 = 0	
26	Albumin	≥40 = 0; <40 and ≥35 = 0.5; <35 = 1.0	
27	Neutrophil/lymphocyte count ratio	≥ 3 = 1.0; <3 = 0	
**Other aspects**			
28	Multiple medications (number of long‐term medications ≥5)	1.0	0

IrAEs were defined as (1) pathological evidence of irAEs, (2) multidisciplinary judgment including at least two oncological and respiratory specialists, or (3) clinical improvement after treatment based on clinical and radiographic findings of irAEs.^14^, ^15^, ^16^ The severity of irAEs was graded according to the Common Terminology Criteria for Adverse Events (CTCAE) 5.0 criteria. Additionally, the duration of PD‐1 inhibitor treatment for a patient in the medical record was defined as the time between the initiation of PD‐1 inhibitor treatment and any events leading to treatment discontinuation. Clinical evaluation was performed according to the Response Evaluation Criteria for Solid Tumors, version 1.1 (RECIST 1.1), and computed tomography was performed every 6–8 weeks for response evaluation.^17^

### 2.2 Statistical analysis

Skewed distributions of continuous variables were expressed as median (inter‐quartile range [IQR]), and normal distributions were expressed as mean ± standard deviation (SD). Student's *t*‐test was used for continuous variables with normal distribution, while the Mann–Whitney test was used for continuous variables with non‐normal distribution. The Chi‐square or Fisher's exact tests were used to compare categorical variables between non‐frail and frail patients. *p*‐values < 0.05 were considered statistically significance. All statistical analyses were performed using SPSS (version 23.0). This study's figures were produced using GraphPad Prism 8.0.

## 3 RESULTS

### 3.1 Baseline characteristics

In this study, 114 patients with advanced lung cancer were enrolled, including 104 (91.2%) patients with non‐small cell lung cancer (NSCLC) and 10 (8.8%) patients with small‐cell lung cancer (SCLC). Table 2 displays the baseline characteristics of the 114 patients with lung cancer treated with a PD‐1 inhibitor. The median age was 66 years old. The male‐to‐female ratio was 4.7:1. The median lymphocyte count was 1.1 × 10^9^/L. Thirty‐two patients (28%) with ECOG performance status = 0, 59 patients (51.8%) with ECOG performance status = 1, 15 patients (13.2%) with ECOG performance status = 2, and eight patients with ECOG performance status ≥3. Sintiliumab (*n* = 48) was the most widely used PD‐1 inhibitor, followed by Camrelizumab (*n* = 39), Tislelizumab (*n* = 24), and Pembrolizumab (*n* = 3). More than half of the patients (63.2%) received PD‐1 inhibitor plus chemotherapy, 12 received immunotherapy combined with chemotherapy and targeted therapy, and nine received immunotherapy plus targeted therapy.

**TABLE 2:** Baseline characteristics.

	Total (*n* = 114)	Non‐frail patients (*n* = 75)	Frail patients (*n* = 39)	*p*‐value
Age, median (IQR), year	66 (60,70)	63 (58，69)	70 (65,73)	<0.01
Sex, *n* (%)				0.49
Male	94 (82.5)	60 (80.0)	34 (87.2)	
Female	20 (17.5)	15 (20.0)	5 (12.8)	
ECOG performance status, *n* (%)				<0.01
0	32 (28.0)	27 (36.0)	5 (12.8)	
1	59 (51.8)	43 (57.3)	16 (41.0)	
2	15 (13.2)	5 (6.7)	10 (25.6)	
≥3	8 (7.0)	0 (0)	8 (20.5)	
Tumor type, *n* (%)				0.52
NSCLC	104 (91.2)	67 (89.3)	37 (94.9)	
SCLC	10 (8.8)	8 (10.7)	2 (5.1)	
Lymphocyte counts (×10^9^/L)	1.1 (0.8, 1.5)	1.2 (0.8, 1.6)	1.0 (0.7, 1.3)	0.12
Type of immune checkpoint inhibitor, *n* (%)				0.31
Sintilimab	48 (42.1)	36 (48.0)	12 (30.8)	
Camrelizumab	39 (34.2)	23 (30.7)	16 (41.0)	
Tislelizumab	24 (21.1)	14 (18.6)	10 (25.6)	
Pembrolizumab	3 (2.6)	2 (2.7)	1 (2.6)	
Combination medication, *n* (%)				0.32
Chemotherapy	72 (63.2)	48 (64.0)	24 (61.5)	
Targeted therapy	9 (7.9)	4 (5.3)	5 (12.8)	
Chemotherapy + Targeted therapy	12 (10.5)	10 (13.3)	2 (5.1)	

Abbreviations: NSCLC, non‐small cell lung cancer; SCLC, small cell lung cancer.

### 3.2 Frailty scores

Seventy‐five patients (65.8%) had a FI < 0.25 and were classified as non‐frail, and 39 patients (34.2%) had a FI ≥0.25 and were classified as frail. The non‐frail and frail groups had average ages of 63 and 70 years, respectively (*p* < 0.01). The frailty group had a higher ECOG PS (*p* < 0.01) than the non‐frailty group. Gender, tumor type, lymphocyte count, PD‐1 inhibitor type, and treatment regimen had no significant differences.

### 3.3 Immune‐related adverse events in non‐frail and frail patients

Table 3 depicts the occurrence of irAEs in the non‐frail and frail patients. Twenty patients (17.5%) experienced irAEs, including seven with grade ≥ 3 irAEs. Frail patients had a slight increase in irAEs (14.7% vs. 23.1%, *p* = 0.26) or ≥3 irAEs (5.3% vs. 7.7%, *p* = 0.93), but there was no significant difference between non‐frail and frail patients. Frailty patients were more likely to experience multiple types of irAEs than non‐frailty patients once irAEs occurred (*p* < 0.01). Twenty patients developed irAEs, 16 patients discontinued the use of PD‐1 inhibitors, and 13 patients required corticosteroid therapy, with mainly presented with pneumonitis (11.4%), myocarditis (2.6%), dermatitis (1.7%), neurologic toxicity (2.6%), hepatitis (0.9%), thyroid dysfunction (0.9%), cystitis (0.9%), and renal insufficiency (0.9%). Checkpoint inhibitor pneumonitis (CIP) is the most common PD‐1‐related adverse effect in lung cancer patients. Frail patients are more likely to have CIP than non‐frail patients (20.5% vs. 6.7%, *p* = 0.03).

**TABLE 3:** Summary of immune‐related adverse events.

	Total (*n* = 114)	Non‐frail patients (*n* = 75)	Frail patients (*n* = 39)	*p*‐value
IrAEs, *n* (%)	20 (17.5)	11 (14.7)	9 (23.1)	0.26
Grade ≥ 3 irAEs, *n* (%)	7 (6.1)	4 (5.3)	3 (7.7)	0.93
>1 type of irAEs	5 (4.4)	0 (0)	5 (12.8)	<0.01
Type of clinically relevant irAE, *n* (%)				
Pneumonitis	13 (11.4)	5 (6.7)	8 (20.5)	0.03
Non‐pneumonitis	12 (16)	6 (8)	6 (15.4)	0.22
Hepatitis	1 (0.9)	1 (1.3)	0 (0)	
Dermatitis	2 (1.7)	1 (1.3)	1 (2.6)	
Myocarditis	3 (2.6)	2 (2.7)	1 (2.6)	
Thyroid dysfunction	1 (0.9)	1 (1.3)	0 (0)	
Cystitis	1 (0.9)	0 (0)	1 (2.6)	
Renal insufficiency	1 (0.9)	0 (0)	1 (2.6)	
Neurologic toxicity	3 (2.6)	1 (1.3)	2 (5.1)	
Time to onset of irAEs (days)	74.5 (47.3, 175.8)	80 (34, 114)	122.5 (53, 250)	0.41
Use of corticosteroids, *n* (%)	13 (11.4)	7 (9.3)	6 (15.4)	0.34
Treatment discontinuation due to irAEs, *n* (%)	16 (14.0)	9 (12.0)	7 (17.9)	0.39
Hospitalization due to irAEs, *n* (%)	9 (7.9)	4 (5.3)	5 (12.8)	0.30
Duration of hospitalization (days)	3 (1, 8)	3 (1, 7)	6 (2, 11)	0.01
ICU admission, *n* (%)	2 (1.7)	0 (0)	2 (5.1)	0.12

Abbreviation: irAEs, immune‐related adverse events.

**FIGURE 1:**
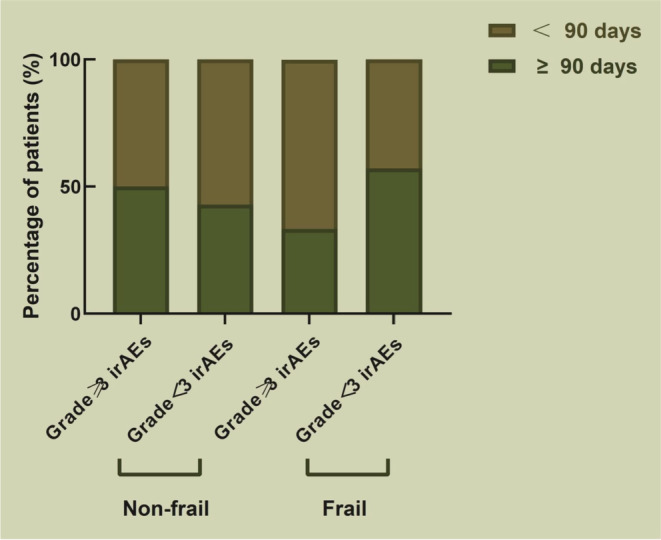
The treatment period for patients with irAEs of varying severity. Frail and non‐frail patients were divided into ≥3 irAEs and <3 irAEs. Among the frail patients, 33.3% (1/3) patients ≥3 irAEs and 57.1% (4/7) patients <3 irAEs could continue treatment with PD‐1 inhibitors for ≥90 days; among the non‐frail patients, 50% (2/4) patients ≥3 irAEs and 42.9% (3/7) patients <3 irAEs could continue treatment with PD‐1 inhibitors for ≥90 days. There was no statistically significant difference between the two subgroups (*p* > 0.05).

When we compared the treatment duration between the two groups, patients in the frailty group was treated with PD‐1 inhibitors until irAEs appeared that the median duration was longer (122.5 vs. 80, *p* = 0.41), although it was not statistically significant. When we focused on irAEs severity and the duration of PD‐1 inhibitor treatment, patients with grade < 3 irAEs in the frailty group displayed a tendency to continue treatment for ≥90 days, but there was no statistically significant difference (*p* > 0.05) between frail and non‐frail patients (Figure 1). Frail patients were more likely than non‐frail patients to discontinue PD‐1 inhibitor therapy (17.9% vs. 12%, *p* = 0.39) or to use corticosteroid therapy (15.4% vs. 9.3%, *p* = 0.34) and had a higher rate of hospitalization (12.8% vs. 5.3%, *p* = 0.30) and ICU admission (5.1% vs. 0%, *p* = 0.12) due to irAEs, even if there were no statistically significant differences. Meanwhile, frail patients had a longer median hospital stay than non‐frail patients (6 vs. 3 days, *p* = 0.01).

### 3.4 Development of IrAEs and clinical outcomes for patients

Tables 4 and 5 demonstrate the development of irAEs and clinical outcomes for non‐frail and frail patients. Frail and non‐frail patients were similar; in both cases, two patients were treated with a PD‐1 inhibitor, and the remaining patients were treated with a PD‐1 inhibitor combined with chemotherapy. Five non‐frail patients were with grade 1 irAEs, two with grade 2 irAEs, and four with grade 3 irAEs. The grade ≥ 3 irAEs occurred in four patients treated with PD‐1 inhibitor combined with chemotherapy. Four frail patients were with grade 1 irAEs, three with grade 2 irAEs, and three with grade 3 irAEs. The grade ≥ 3 irAEs occurred in one patient treated with a PD‐1 inhibitor combined with chemotherapy and two treated with a PD‐1 inhibitor. All non‐frail patients (including four patients with ≥3 irAEs, one with grade 1 dermatitis, and one with grade 2 CIP) who were reactivated with the same PD‐1 inhibitor recovered from irAEs. In contrast, one patient was admitted to the ICU with CIP in frail patients (Table 5. Case 6), and the remaining patients recovered from irAEs. Two patients returned to the same PD‐1 inhibitor therapy, including one patient with NSCLC who developed grade 2 dermatitis, retreated with the same PD‐1 inhibitor, and developed CIP after 259 days (Table 5. Case 8). Another patient with grade 1 CIP was restarted with the same PD‐1 inhibitor with no other adverse events (Table 5. Case 1). Patients were followed up for 30, 60, 90, and 120 days after the diagnosing irAEs. Four patients were lost to follow‐up, and none of the others died.

**TABLE 4:** Clinical features of the non‐frail patients with irAEs

Case	Age	Sex	Tumor type	Type of immune checkpoint inhibitors		IrAEs	Grade	Time to onset of irAEs (days)	Remission of irAEs	Restart of PD‐1 treatment	Follow‐up time (days)			
					Chemotherapy						30	60	90	120
1	68	F	SCLC	Sintilimab	No	Myocarditis	1	48	Yes	No	Yes	Yes	Yes	Yes
2	69	M	NSCLC	Tislelizumab	Yes	Dermatitis	1	1	Yes	Yes	Yes	Yes	Yes	Yes
3	66	M	NSCLC	Sintilimab	Yes	Myocarditis	3	56	Yes	No	Yes	Yes	Loss	Loss
4	64	M	NSCLC	Tislelizumab	Yes	Interstitial pneumonitis	2	32	Yes	Yes	Yes	Yes	Yes	Yes
5	70	M	NSCLC	Camrelizumab	Yes	Interstitial pneumonitis	1	104	Yes	No	Yes	Yes	Yes	Yes
6	67	M	SCLC	Sintilimab	Yes	Hepatitis	1	80	Yes	No	Yes	Yes	Loss	Loss
7	62	F	NSCLC	Camrelizumab	Yes	Interstitial pneumonitis	3	175	Yes	No	Yes	Yes	Yes	Yes
8	50	M	SCLC	Sintilimab	Yes	Interstitial pneumonitis	3	34	Yes	No	Yes	Yes	Yes	Yes
9	50	M	SCLC	Sintilimab	Yes	Thyroid dysfunction	2	209	Yes	No	Loss	Loss	Loss	Loss
10	58	M	NSCLC	Sintilimab	No	Interstitial pneumonitis	1	114	Yes	No	Loss	Loss	Loss	Loss
11	59	M	NSCLC	Camrelizumab	Yes	Neurologic toxicity	3	95	Yes	No	Yes	Yes	Yes	Yes

Abbreviations: irAEs, immune‐related adverse events; NSCLC, non‐small cell lung cancer; SCLC, small‐cell lung cancer.

**TABLE 5:** Clinical features of the frail patients with irAEs

Case	Age	Sex	Tumor type	Type of immune checkpoint inhibitors	Chemotherapy	IrAEs	Grade	Time to onset of irAEs(days)	Remission of irAEs	Restart of PD‐1 treatment	Follow‐up time (days)			
											30	60	90	120
1	68	M	NSCLC	Camrelizumab	Yes	Interstitial pneumonitis	1	346	Yes	Yes	Yes	Yes	Yes	Yes
2	65	M	NSCLC	Sintilimab	Yes	Interstitial pneumonitis;	2	176	Yes	No	Yes	Yes	Yes	Yes
						Neurologic toxicity	2		Yes					
3	77	M	NSCLC	Tislelizumab	Yes	Interstitial pneumonitis	2	47	Yes	No	Yes	Yes	Yes	Yes
4	61	M	NSCLC	Camrelizumab	Yes	Interstitial pneumonitis	3	214	Yes	No	Yes	Yes	Yes	Yes
5	79	M	NSCLC	Tislelizumab	Yes	Interstitial pneumonitis	1	55	Yes	No	Yes	Yes	Loss	Loss
						Myocarditis	1		Yes					
6	73	M	NSCLC	Sintilimab	No	Interstitial pneumonitis	3	69	No	No	Loss	Loss	Loss	Loss
7	60	M	NSCLC	Sintilimab	No	Neurologic toxicity;	2	65	Yes	No	Yes	Yes	Yes	Yes
						Adrenal insufficiency	3		Yes					
8	64	M	NSCLC	Camrelizumab	Yes	Dermatitis;	2	22	Yes	Yes	Yes	Yes	Yes	Yes
						Interstitial pneumonitis	1	325	Yes	No				
9	75	M	SCLC	Pembrolizumab	Yes	Interstitial pneumonitis;	1	225	Yes	No	Yes	Yes	Yes	Yes
						Cystitis	1		Yes					

Abbreviations: irAEs, immune‐related adverse events; NSCLC, non‐small cell lung cancer; SCLC, small‐cell lung cancer.

## 4 DISCUSSION

In this retrospective study, we employed 28‐item FI to assess the effect of frailty on PD‐1 inhibitor‐related adverse events in patients with advanced progressive lung cancer. According to our knowledge, this is the first study to evaluate the association between 28 indirect markers of frailty and clinical outcomes in advanced lung cancer patients treated with PD‐1 inhibitors. Fan et al.^13^ demonstrated that 28‐item FI was associated with all‐cause and cause‐specific mortality, independent of chronological age, in younger and elderly Chinese. It may help to prevent premature deaths, and extend healthy and positive life expectancy using alternative measures to identify younger adults suffering from accelerated aging. According to FI developed by Fan et al., we collected 28 indicators, including 15 underlying diseases, three types of functional disorders, five types of independent living ability, four kinds of physiological indicators, and one other condition to evaluate lung cancer patients. The FI was used in younger adults and older patients.

Several studies have investigated the association between frailty and the occurrence of irAEs assessed by scoring tools other than the 28‐item FI. A retrospective study of 197 patients with advanced malignant tumors (including 77 patients with NSCLC) using indirect markers of frailty, such as ECOG performance status, Charlson Comorbidity Index (CCI), and NLR, discovered that frail patients in the elderly and non‐elderly groups have no association with irAEs or complications.^18^ Kubo et al.^19^ retrospectively studied the safety of ICIs in 95 patients ≥75 years with NSCLC and calculated a modified G8. They concluded that impaired, modified G8 was not associated with grade ≥ 2 irAEs. Welaya et al.^20^ performed a small retrospective study of 28 patients, 11 of whom had lung cancer, and found no association between Geriatric assessment (GA) impairment and the development of irAEs. We did not identify 28‐item studies on the relationship between FI and the occurrence of irAEs.

Our data revealed that the incidence of irAEs was 17.5%, of which the grade ≥ 3 irAEs was 6.1% and the CIP was 11.4%. The results are lower than clinical studies reported in the literature.^21^, ^22^, ^23^, ^24^, ^25^ Frail patients developed slightly more irAEs of any grade with PD‐1 inhibitors than non‐frail patients, although this difference was not statistically significant. Specifically, the most common adverse effect was CIP, which was more common in frail patients. Other irAEs included myocarditis, neurologic toxicity, dermatitis, thyroid dysfunction, hepatitis, cystitis, and renal insufficiency, but no significant differences were observed. Recently, pulmonary adverse events caused by PD‐1 inhibitors, including some severe and even fatal events, have been reported.^26^ CIP accounted for 35% of deaths associated with PD‐1 and PD‐L1 inhibitors. Furthermore, the incidence of CIP caused by PD‐1 inhibitors was higher in NSCLC patients than in other cancer patients.^27^, ^28^ This will make clinicians more cautious when prescribing PD‐1 inhibitors to frail lung cancer patients.

In our study, all patients with irAEs recovered with appropriate treatment in non‐frail patients, and the same PD‐1 inhibitor was restarted in cases with grade < 3 irAEs without other adverse effects. In contrast, although most patients recovered after treatment in the frail group, there was a recurrence of adverse effects in patients with <3 irAEs after the reintroduction of the same PD‐1 inhibitor. There are conflicting reports on the safety and efficacy of retreatment after irAEs, and no consensus has been reached. Therefore, restarting PD‐1 inhibitors after irAEs may be challenging for frailty patients.^29^, ^30^, ^31^ Additionally, we discovered that frail patients were less likely to have ≥3 irAEs with the longer duration of continuous use of PD‐1 inhibitors until the irAEs occur, although the development of severe irAEs in frail patients is similar to that of non‐frail patients. It indicates that PD‐1 inhibitors may have a certain degree of efficacy and tolerance in lung cancer patients. Surprisingly, our study showed that frail patients had longer hospital stays, and irAEs were presented when they had more than one type of irAEs. This supports the importance of frailty for developing irAEs in lung cancer patients treated with PD‐1 inhibitors. Multiple adverse events should be considered when frail patients develop irAEs after receiving PD‐1 inhibitors.

This study has inherent limitations as a retrospective study. First, the sample size of patients was relatively small. Second, some patients with milder symptoms of grade 1–2 irAEs were excluded from the study because they were frequently observed in the outpatient department rather than hospitalized. Neglecting such patients, especially skin and other adverse events, may lead to underestimating the incidence. Finally, we did not exclude patients with inadequate medical records, for example, who were lost to follow‐up after 60 days to document as many irAEs as possible.

In conclusion, although this study does not provide sufficient evidence that frailty is associated with the incidence of total irAEs or grade ≥ 3 irAEs according to FI, frailty is associated with CIP. Moreover, once irAEs occur in frail patients, it is often manifested by more than one adverse event. Assessing the risk of frailty by FI can help identify those frail patients at risk of CIP and predict the length of hospital stay. Therefore, FI assessment in patients receiving PD‐1 inhibitors is feasible and should be considered. Ultimately, a deeper understanding of a patient's frailty can serve as a guide to making personalized treatment decisions.

## AUTHOR CONTRIBUTIONS

**Jun Li:** Data curation (equal); formal analysis (lead); methodology (equal); software (equal); writing – original draft (lead). **Xiaolin Zhang:** Conceptualization (equal); funding acquisition (equal); methodology (equal); project administration (lead); writing – review and editing (lead). **Shuang Zhou:** Investigation (equal); resources (equal). **Ying Zhou:** Investigation (equal); resources (equal). **Xinmin Liu:** Project administration (equal); supervision (lead).

## FUNDING INFORMATION

This work was supported by the National Key R&D Program of China (No. 2020YFC2008804).

## CONFLICT OF INTEREST STATEMENT

All authors declared no potential conflicts of interest with respect to the research, authorship, and/or publication of this article.

## Data Availability

Data available on request from the authors.
